# Two formulations of coronavirus disease‐19 recombinant subunit vaccine candidate made up of S1 fragment protein P1, S2 fragment protein P2, and nucleocapsid protein elicit strong immunogenicity in mice

**DOI:** 10.1002/iid3.748

**Published:** 2022-11-25

**Authors:** Erkan Özcengiz, Duygu Keser, Gülay Özcengiz, Gözde Çelik, Aykut Özkul, Fatma Nur İnçeh

**Affiliations:** ^1^ Vaccine R&D Pharmada Pharmaceuticals Ankara Turkey; ^2^ Department of Biological Sciences Middle East Technical University Ankara Turkey; ^3^ Department of Virology, School of Veterinary Medicine Ankara University Ankara Turkey; ^4^ R&D Department Kobay A.Ş. Ankara Turkey

**Keywords:** COVID‐19, nucleocapsid protein, receptor binding domain (RBD), recombinant subunit vaccine, SARS‐CoV‐2, spike protein

## Abstract

**Introduction:**

Coronavirus disease (COVID‐19) is ongoing as a global epidemic and there is still a need to develop much safer and more effective new vaccines that can also be easily adapted to important variants of the pathogen. In the present study in this direction, we developed a new COVID‐19 vaccine, composed of two critical antigenic fragments of the S1 and S2 region of severe acute respiratory syndrome coronavirus 2 as well as the whole nucleocapsid protein (N), which was formulated with either alum or alum plus monophosphoryl lipid A (MPLA) adjuvant combinations.

**Methods:**

From within the spike protein S1 region, a fragmented protein P1 (MW:33 kDa) which includes the receptor‐binding domain (RBD), another fragment protein P2 (MW:17.6) which contains important antigenic epitopes within the spike protein S2 region, and N protein (MW:46 kDa) were obtained after recombinant expression of the corresponding gene regions in *Escherichia coli* BL21. For use in immunization studies, three proteins were adsorbed with aluminum hydroxide gel and with the combination of aluminum hydroxide gel plus MPLA.

**Results:**

Each of the three protein antigens produced strong reactions in enzyme‐linked immunosorbent assays and Western blot analysis studies performed with convalescent COVID‐19 patient sera. In mice, these combined protein vaccine candidates elicited high titer anti‐P1, anti‐P2, and anti‐N IgG and IgG2a responses. These also induced highly neutralizing antibodies and elicited significant cell‐mediated immunity as demonstrated by enhanced antigen‐specific levels of interferon‐γ (INF‐γ) in the splenocytes of immunized mice.

**Conclusion:**

The results of this study showed that formulations of the three proteins with Alum or Alum + MPLA are effective in terms of humoral and cellular responses. However, since the Alum + MPLA formulation appears to be superior in Th1 response, this vaccine candidate may be recommended mainly for the elderly and immunocompromised individuals. We also believe that the alum‐only formulation will provide great benefits for adults, young adolescents, and children.

## Graphical abstract

Our study describes the development of two different formulations of a recombinant multisubunit vaccine against the severe acute respiratory syndrome coronavirus 2 using an *Escherichia* coli expression system, as well as preclinical testing with these formulations.

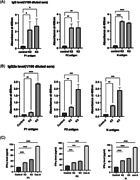

## 1 INTRODUCTION

Severe acute respiratory syndrome coronavirus 2 (SARS‐CoV‐2) is a global threat due to its highly pathogenic and invasive nature and has caused the deaths of millions of people worldwide over the past 2 years. Still today, the coronavirus disease (COVID‐19) pandemic continues threatening lives and raises serious global concerns.

Coronaviruses are spherical, enveloped viruses with genomes consisting of single‐stranded positive‐sense RNA (+ssRNA) of approximately 30 kb with a 5′‐cap and 3′‐poly‐A tail. The genome of a typical CoV contains six or more open reading frames (ORFs). The first ORF (ORF1a/b) covers approximately 66% of the entire genome and encodes 16 nonstructural proteins (nsp1–16) which are mainly involved in viral replication. Covering one‐third of the genome near the 3′‐terminus, the other ORF codes for spike (S), membrane (M), envelope (E), and nucleocapsid (N) proteins which are the main structural proteins necessary for the formation of the virion and its infectious capacity.^1^ The homotrimers of the S glycoprotein form spikes on the viral surface and are responsible for binding to the host cell receptors. The high contagiousness of the virus is due to the high affinity of this protein for the angiotensin‐converting enzyme 2 (ACE‐2) receptor.^2^ The M protein contains three transmembrane domains and covers the nucleocapsid, giving shape to the virion and supporting membrane curvature. The E protein participates in virus aggregation and release and is also involved in viral pathogenesis. The N protein has two domains that bind to the viral genome, and it also counteracts the antiviral effects of interferons (INFs).^3^

The severity of the immune response against the structural antigens of SARS‐CoV‐2 is significant for recovery from the disease. The S protein initiates the fusion of viral and host membranes and is the main antigenic component among the structural proteins of the pathogen and neutralizing antibodies (nAbs) targeting the receptor‐binding domain (RBD) of the S1 subunit induce protective immunity against infection.^4^ For this reason, it has been the focus of vaccine design. On the other hand, it was reported that T cell responses to S and N proteins are the most prolonged.^5^, ^6^, ^7^, ^8^ For long‐term immune protection, there is potential to prevent recurrent COVID‐19. T cell responses against the structural N‐protein and nonstructural NSP7 and NSP13 encoded by ORF1 were studied by Le Bert et al.^9^ in a total of 36 patients convalescing from COVID‐19. In all these patients, CD4 and CD8 cells that recognized multiple regions of the N protein were found. Also, long‐lasting memory T cells that are reactive to the N protein of SARS‐CoV of the 2003s outbreak were also shown in 23 patients who recovered 17 years ago, displaying robust cross‐reactivity of the N protein of SARS‐CoV‐2.

In the present study, with the aim of developing an effective and safe COVID‐19 vaccine, two critical antigenic fragments of the S protein and the whole N protein of SARS‐CoV‐2 were obtained by recombinant DNA technology and formulated with either alum or alum plus monophosphoryl lipid A (MPLA) adjuvant combinations. These formulations were shown to be highly immunogenic and produced nAbs against SARS‐CoV‐2 in animal experiments.

## 2 MATERIALS AND METHODS

### 2.1 Bacterial strains and plasmids

*Escherichia coli* DH5α (ATCC) and *E. coli* BL21(DE3) (Novagen) were the bacterial hosts for cloning and expression studies, respectively. The vectors pGEM‐T Easy (Promega) and pET28a(+) (Novagen) were used for polymerase chain reaction (PCR) cloning and expression, respectively.

### 2.2 Molecular cloning of P1, P2, and N genes and expression of recombinant P1, P2, and N proteins

The ORF of the p1 and p2 genes were amplified from Full Spike (S) Expression Vector‐Cat. Code:pUNO1‐SARS2‐S, Invivogen by PCR using specific primers designed with *NheI* and *BamHI* restriction sites for P1 and *NheI* and *SacI* (underlined) restriction sites for P2. The N gene was amplified from Nucleocapsid Expression Vector‐Cat. Code:pUNO1‐SARS2‐N–Invivogen by PCR using specific primers designed with *NheI* and *BamHI* restriction sites (underlined).

The primers for PCR cloning were tabulated in Table 1.

**Table 1:** Reverse and forward primers for PCR cloning

Name	Target gene	Nucleotide sequence (5′–3′)	Length (bp)	Size of PCR product
P1 forward primer	Spike/S1	5′‐CCTCTCTCAGAAGCTAGCTGTACGTTGAA‐3′	29	819 bp
P1 reverse primer		5′‐ACGGACGGATCCTTAAGTAGTGTCAGCA‐3′	28	
P2 forward primer	Spike/S2	5′‐TTAGCGGGTACAATCGCTAGCGGTTGGACCTTTGG‐3′	35	489 bp
P2 reverse primer		5′‐ ACTGAGGGAAGGAGAGCTCTTAATAGCCCTTTCC‐3′	34	
N forward primer	Nucleocapsid	5′‐CTGAGATCACCGGCTAGCATGTCTGATAATGG‐3′	32	1257 bp
N reverse primer		5′‐ATGTCTGGCCAGGGATCCTTAGGCCTGAGTT‐3′	31	

Abbreviation: PCR, polymerase chain reaction.

After PCR, the products were cloned into pGEM®‐T Easy in *E*.*coli* DH5α and then into the *BamHI* restriction site of expression vector pET‐28a(+)(pET28–P1, pET28‐P2, pET28‐N) in *E. coli* BL21(DE3).

The genes were subsequently cloned into pET28a to express them as His‐tagged proteins (pET28‐P1, pET28‐P2, and pET28‐N).

For expression of recombinant P1, P2, and N proteins, *E. coli* BL21(DE3) cells containing pET28–P1, pET28‐ P2, and pET28‐N, respectively, were incubated in 10 ml of Luria Broth (LB) broth (Merck) containing 30 mg/ml kanamycin (Sigma) overnight by shaking at 37°C, after which 3 ml of each culture was transferred into 200 ml of LB containing kanamycin. When OD_600_ of the culture had reached 0.6, protein expression was induced by the addition of isopropylβ‐D‐1‐thiogalactopyranoside (IPTG; Sigma) at a final concentration of 1 mM. The culture was incubated for a further 5 h after the addition of IPTG to obtain the necessary amount of recombinant P1, P2, and N proteins, respectively.

### 2.3 Purification of recombinant P1, P2, and N proteins

After expression of the rP1, P2, and N proteins via IPTG induction in *E. coli* BL21(DE3), the cells were harvested by centrifugation and resuspended in denaturing solubilization buffer (DSB) containing 50 mM NaH_2_PO_4_, 0.3 M NaCl, and 8 M urea (pH 8.0). The suspension was kept at −80°C for 15 min, thawed, vortexed twice, and then lysed by sonication using a CP70T Ultrasonic Processor(Cole‐Parmer) for 6–10 s at 60% amplitude. After centrifugation, the supernatant containing the protein of interest was collected. The recombinant proteins were purified with TAKARA® His60 Nickel affinity columns. The columns were equilibrated with 4 ml of DSB, and the supernatant was loaded after the columns were washed three times with DSB. Each protein was eluted with 3 ml denaturing elution buffer containing 50 mM NaH_2_PO_4_, 0.3 M NaCl, 8 M urea, and 250 mM imidazole (pH 8.0). To determine the total purified protein concentration, the modified Bradford assay described by Ramagli and Rodrigez was used.^10^ The purity and immunogenicity of each sample were determined by sodium dodecyl‐sulfate (SDS‐PAGE) and Western blot (WB) analysis, respectively.

### 2.4 WB analysis

Proteins were separated using SDS‐PAGE, then transferred into a nitrocellulose membrane using a horizontal semidry blotter (Cleaver Scientific Ltd.). The membrane was blocked at 37°C with 10% skim milk, after the transfer. After blocking, the membrane was incubated with the primary antibody, which was either sera from vaccinated mice or convalescent COVID‐19 patients were prepared as a 1/300 dilution in 5% skim milk. Then the membrane was incubated with secondary antibodies which are rabbit anti‐mouse IgG‐alkaline phosphatase (Sigma), at a dilution of 1/10,000 or goat antihuman IgG‐alkaline‐phosphatase (AP) (Southern Biotech), at a dilution of 1/3000. Then the membrane was incubated with AP conjugate substrate (Bio‐Rad) until the protein bands could be seen.

### 2.5 The P1 protein binding activity to ACE2

An enzyme‐linked immunosorbent assay (ELISA) plate was pre‐coated with 100 μl/well of P1 (containing RBD) fragment protein at 2 μg/ml in carbonate buffer (pH 9.6). After overnight incubation plate was washed and blocked with 100 μl/well 2% BSA. Then, 25 ng ACE‐2‐His‐Biotin (BPS Bioscience) protein solution was added at 100 μl/well, then incubated for 1 h at 37°C. Then to detection of color change, biotinylated ACE‐2 was bound with horseradish peroxidase (HRP)‐conjugated streptavidin (BPS Bioscience) and 100 μl/well of colorimetric substrate tetramethylbenzidine (TMB) was added and incubated for 30 min at room temperature; the reaction was stopped with the addition of 50 μl/well 1 N HCl. Optical density was measured at 450 nm with Thermo Scientific™ Multiskan™ FC Microplate Photometer.

### 2.6 Preparation of vaccine formulations and mice immunization

For each group, 10 female BALB/c mice (16–18 g) were immunized intraperitoneally with recP1 (25 µg) + recP2 (25 µg) + recN (25 µg) + Alhydrogel (aluminum hydroxide) (K2) or recP1 (25 µg) + recP2(25 µg) + recN(25 µg) + Alhydrogel + MPLA (PHAD®) (Avanti Polar Lipids Inc.) (K3) combined vaccines and only Alhydrogel + MPLA as a negative control. A second immunization was carried out on Day 15. Blood was collected from mice on Day 30. The sera thus obtained was stored at −20°C until further use. Animal experiments were repeated at least twice.

All animal experiments were performed with the approval of the Ethics Committee on Animal Experimentation, Kobay A.Ş. (Ethical approval number: 508).

### 2.7 Measurement of antibody titers

Specific IgG and IgG2a responses were quantitated by ELISA using sera collected from the vaccinated and the control groups. Purified recombinant P1, P2, and N proteins were used as coating antigens in ELISA at a concentration of 2 μg per well. Murine sera with dilutions 1:100 and 1:1000 were used as primary antibodies. Alkaline phosphatase‐conjugated rat anti‐mouse IgG2a (Southern Biotech) and alkaline phosphatase‐conjugated rabbit anti‐mouse IgG (whole molecule) (Sigma‐Aldrich) were used as the second antibody with the dilution of 1:3000 in blocking solution. p‐nitrophenyl phosphate disodium salt (Thermo Scientific) was used for colorimetric detection. Optical density was measured at 405 nm with Thermo Scientific™ Multiskan™ FC Microplate Photometer.

### 2.8 Detection of antigen‐specific T‐cell response (IFN‐γ)

Two weeks after this immunization, the spleens of five mice from each group were dissected under sterile conditions and transferred to 5 ml of Roswell Park Memorial Institute MediumRPMI 1640 medium containing 10% fetal bovine serum and 1% penicillin/streptomycin (Biochrom). They were then homogenized through a 70 mm nylon cell strainer (BD Bioscience) and cell counting was carried out with a hemocytometer. Splenocytes from the samples were distributed on 96‐well plates at a density of 1 × 10^6^ cells/well in RPMI 1640 medium. The plates were incubated at 37°C in a CO_2_ incubator with 5% CO_2_. After 1 h, the different splenocytes were stimulated with 30 µg/ml of recombinant P1, P2, N, and concanavalin A (1 mg/ml; Sigma). Culture media were collected on the third day (72 h) and analyzed for IFN‐γ using a Mouse IFN‐γ Minikit (Thermo Scientific).

### 2.9 SARS‐CoV‐2 neutralization assay

#### 2.9.1 Microneutralization (MN) assay

The live virus‐based MN assay was used. SARS‐CoV‐2 neutralizing activity was studied in a BSL3 facility at the University of Ankara, School of Veterinary Medicine, as described previously.^11^ Briefly, serum samples diluted 1:5 in cell growth media (High Glucose Dulbecco's modified eagle medium, Gibco GmBH) were mixed with an equal volume of SARS‐CoV‐2 (100TCID_50_ = 10^5.2^/ml) and incubated at 37°C for 60 min. Subsequently, the mixtures were inoculated into Vero E6 cells grown in 24‐well tissue culture plates. Plain culture media was used as the negative control. The neutralizing capacity of each serum dilution was assessed by detecting 100% blockage of SARS‐CoV‐2 CPE (NT100) 4 days postinfection.^11^ The MN titer ≥1/5 was evaluated as positive.

#### 2.9.2 Neutralization of P1 (containing RBD) protein‐ACE2 interaction

An ELISA plate was pre‐coated with 2 μg P1 protein, after blocking, 100 μl of serum samples were added to wells with the dilution of 100, 200, and 400‐fold. And 25 ng ACE‐2‐His‐Biotin (BPS Bioscience) was added to serum samples and incubated at 37°C for 60 min. Then biotinylated ACE‐2 was bound with HRP‐conjugated streptavidin (BPS Bioscience). To observe the colorimetric color change, 3,3′5,5′‐TMB solution (Invivogen), which is the substrate of the HRP enzyme, was used. Optical density was measured at 450 nm with Thermo Scientific™ Multiskan™ FC Microplate Photometer.

### 2.10 Statistical analysis

ELISA results for antibody responses were analyzed by one‐way analysis of variance (ANOVA) followed by Dunnett's multiple comparison test using Graph Pad Prism version 9.3 software program. *p* values < .05 were considered significant for all analyses.

## 3 RESULTS

### 3.1 Purification, identity, and immunogenicity of recombinant proteins

**Figure 1:**
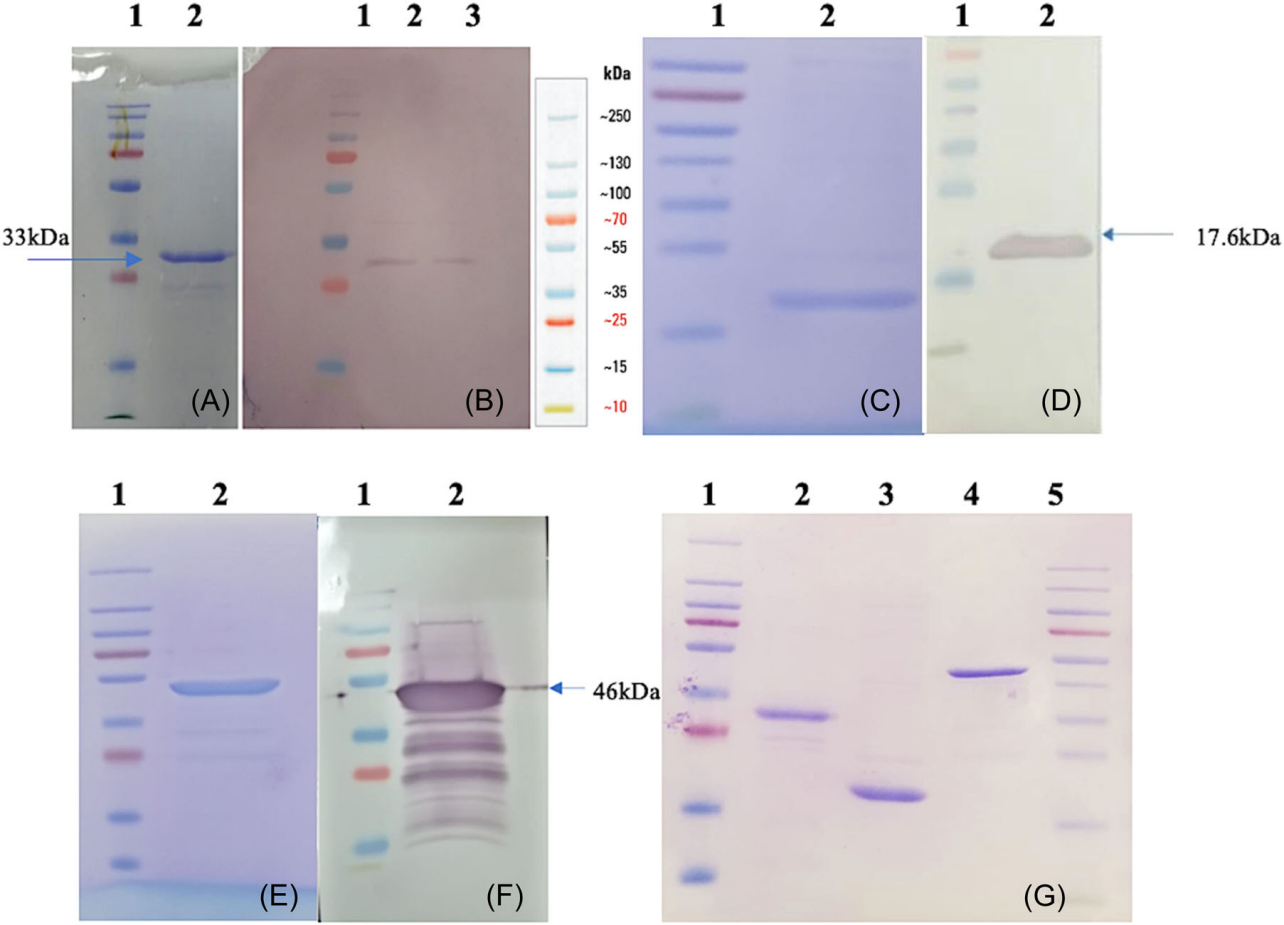
(A) SDS‐PAGE detection of the SARS‐CoV‐2 spike protein S1 region fragment protein (P1:33 kDa) containing RBD; Lane 1, 2: protein ladder and purified P1 protein elute. (B) WB analysis of P1 protein. WB was done using an anti‐Spike‐RBD‐mIgG2a‐monoclonal mouse IgG2a antibody (Invivogen). (C) SDS‐PAGE detection of the SARS‐CoV‐2 spike protein S2 region fragment protein (P2: 17.6 kDa); Lane 1, 2: protein ladder and purified P2 protein elute. (D) Western blot analysis of P2 protein. WB was done using the serum obtained from mice immunized with combined recombinant P1 + P2 + N formulation adjuvanted with alum. (E) SDS‐PAGE detection of the SARS‐CoV‐2 nucleocapsid protein (N; 46 kDa); Lane 1, 2: protein ladder and purified N protein elute. (F) Western blot analysis of N protein. WB was done using an anti‐Nucleocapsid‐mIgG1‐monoclonal mouse IgG1 antibody (Invitrogen). (G) Profiles of purified P1, P2, and N proteins on SDS‐PAGE. RBD, receptor‐binding domain; SARS‐CoV‐2, severe acute respiratory syndrome coronavirus 2; WB, Western blot.

Purified recombinant P1 (33 kDa), P2 (17.6 kDa), and N (46 kDa) proteins were analyzed by SDS‐PAGE after staining with Coomassie blue (Figure 1A,C,E,G). In SDS‐PAGE analyses and WB analyses using specific monoclonal antibodies, anti‐Spike‐RBD‐mIgG2a‐monoclonal mouse IgG2a antibody (Invivogen) and antinucleocapsid‐mIgG1 monoclonal mouse IgG1 antibody (Invitrogen), the pure proteins were specifically determined by their expected molecular sizes (Figure 1B,F). P2 protein was also obtained purely, as shown by SDS‐PAGE and WB analysis using the serum obtained from mice immunized with our combined recP1, P2, and N proteins adjuvanted with alum (Figure 1C,D).

**Figure 2:**
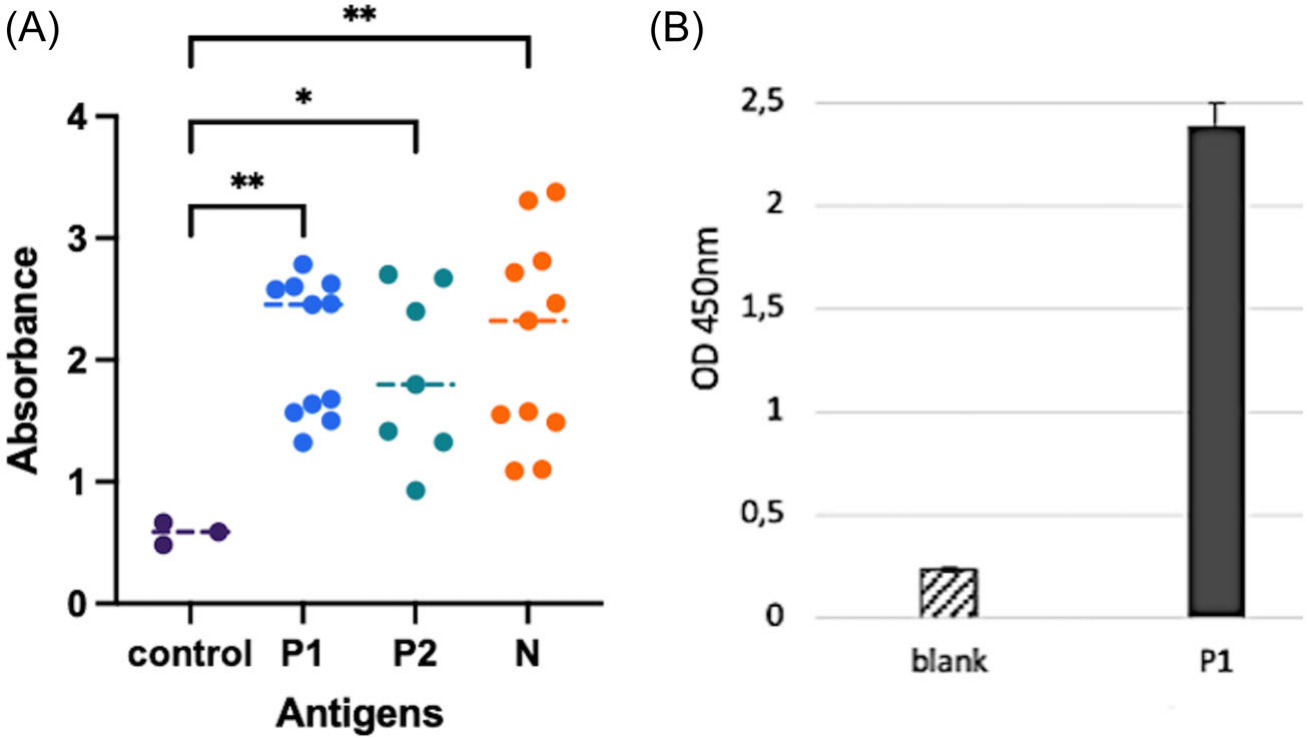
(A) The reaction of the recP1, recP2, and recN antigenic proteins with 1/100 diluted sera was obtained from recovered COVID‐19 patients in ELISA tests. (B) Binding activity between P1 (containing RBD) fragment protein and ACE2. Statistically significant(*p* < .05) increase compared to the control was shown with an asterisk. One‐way ANOVA followed by Dunnett's multiple comparisons test was used for statistical analysis. ACE2, angiotensin‐converting enzyme 2; ANOVA, analysis of variance; COVID‐19, coronavirus disease 19; ELISA, enzyme‐linked immunosorbent assays; RBD, receptor‐binding domain.

**Figure 3:**
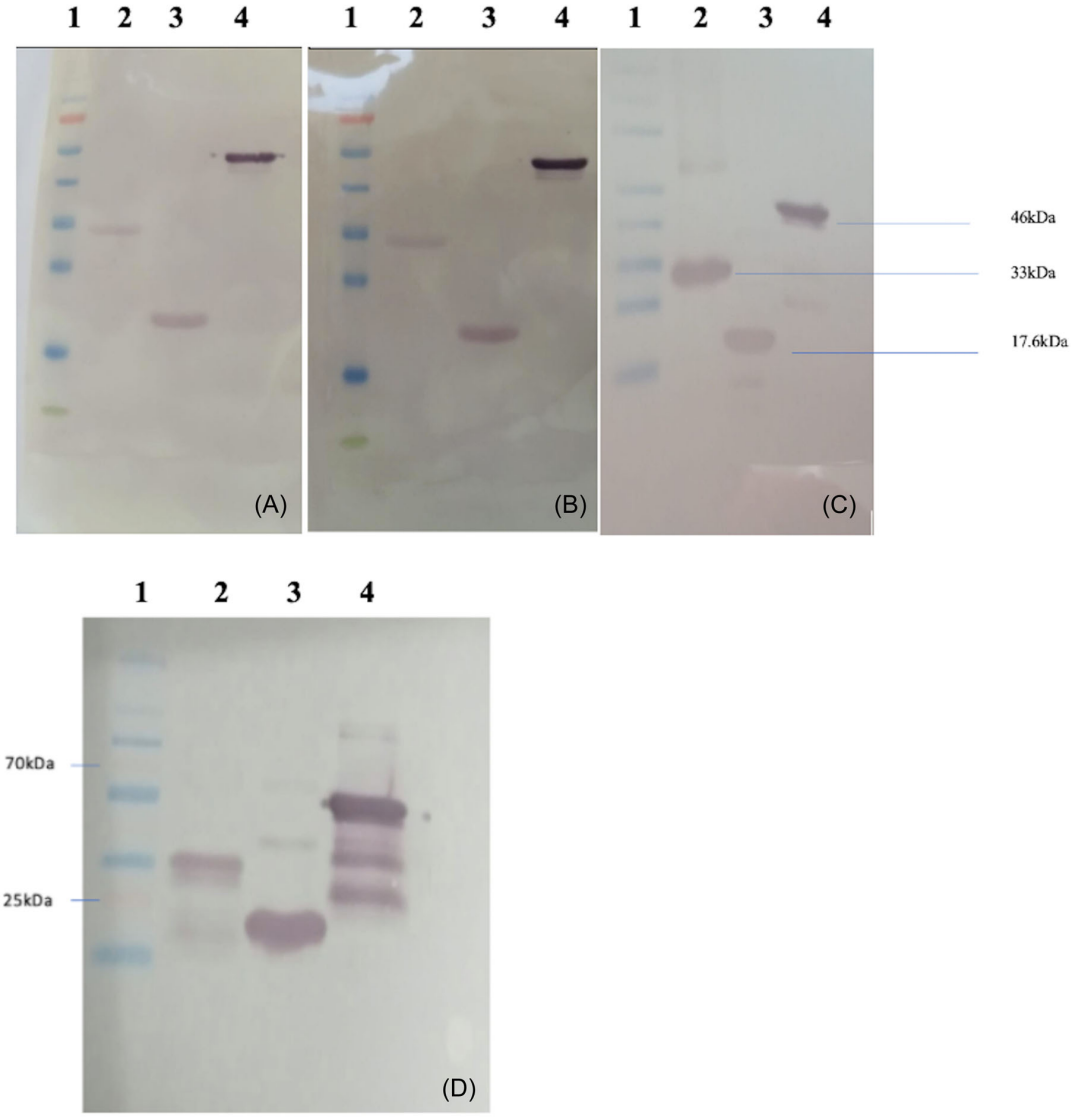
Western blot analysis of SARS‐CoV‐2 recombinant Spike protein S1 and S2 region fragments (P1:33 kDa and P2:17.6 kDa), nucleocapsid protein (pN:46 kDa) using 1/300‐diluted sera from the patients infected with (A) wild type, (B) beta variant, and (C) delta variant SARS‐CoV‐2. Western blot analysis (D) of recombinant P1, P2, and N proteins, using the sera of mice immunized with recombinant P1 + P2 + N combined vaccine formulation adjuvanted with Alhydrogel. Lane 1: protein ladder, lane 2: Purified P1 protein, lane 3: Purified P2 protein, lane 4: Purified N protein. SARS‐CoV‐2, severe acute respiratory syndrome coronavirus 2.

It was determined that each of these three protein antigens gave a strong reaction in ELISA studies performed with convalescent COVID‐19 patient sera (Figure 2A). The P1 protein also demonstrated potently binding activity to ACE2 (Figure 2B). It was also observed that these antigens have a strong reaction in the WB analysis performed with wild‐type, beta variant, and delta variant SARS‐CoV‐2 infected COVID‐19 patient sera (Figure 3A–C).

### 3.2 Immune responses

**Figure 4:**
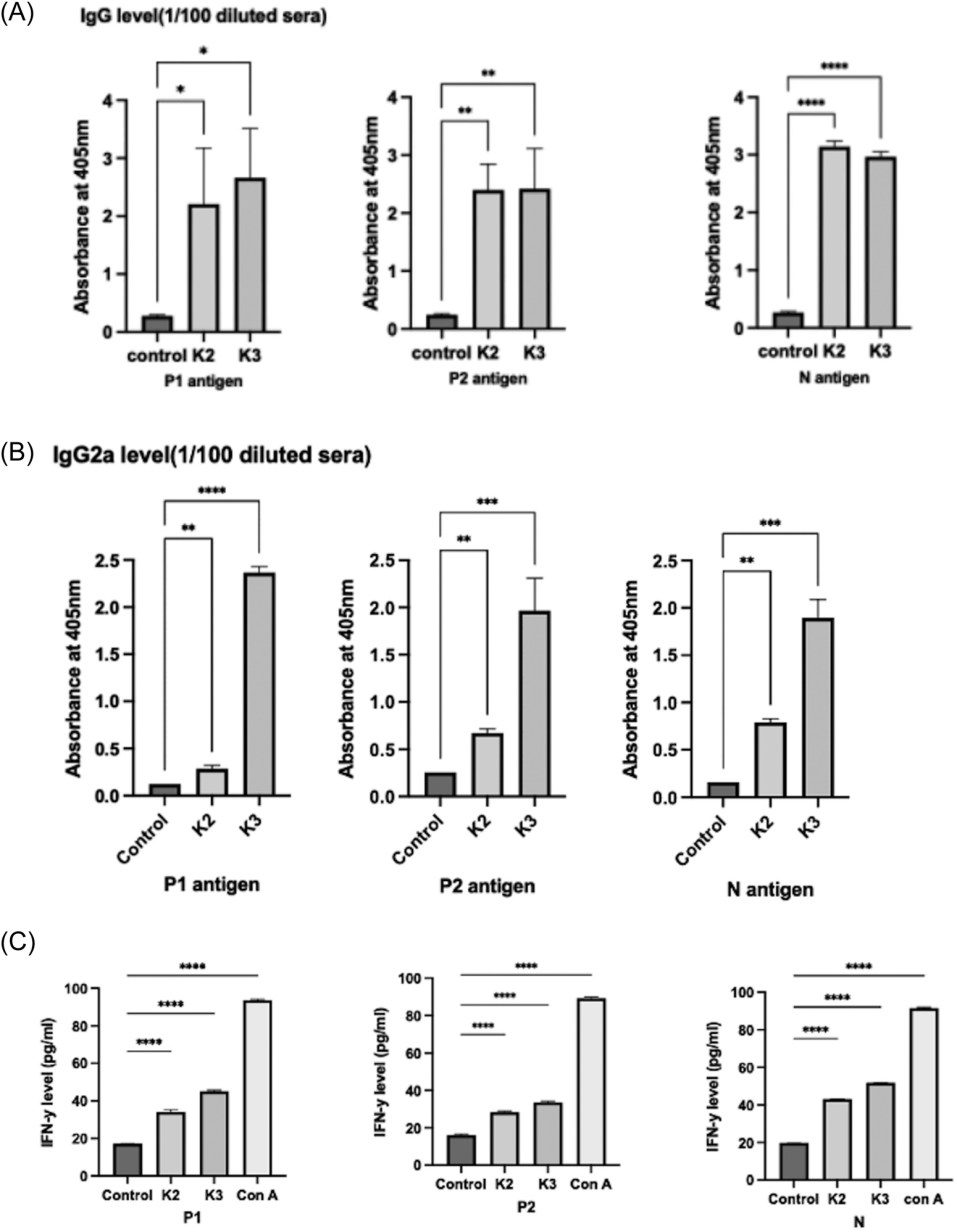
Anti‐P1, anti‐P2, and anti‐N IgG antibody levels (A) and IgG2a antibody levels (B) in mice sera immunized with combined vaccine formulations with Alhydrogel (K2) or Alhydrogel + MPLA (K3) as the adjuvant, respectively. (C) Antigen‐specific IFN‐γ levels as determined in splenocytes of mice immunized with K2 or K3. One‐way ANOVA followed by Dunnett's multiple comparisons test was used for statistical analysis. *p < .05, **p < .01, ***p < .001, ****p < .0001. ANOVA, analysis of variance; ConA, concanavalin A; IFN‐γ, interferon‐γ; MPLA, monophosphoryl lipid A.

The sera of mice immunized with recombinant P1 + P2 + N vaccine formulation adjuvanted with either Alhydrogel or Alhydrogel plus MPLA and nonimmunized control group were collected on Day 14 after this immunization. The strong reaction of all three proteins is seen in the WB analyses using this immune serum (Figure 3D). Figure 4A,B show serum IgG and IgG2a titers, respectively. In immunized mice, serum IgG and IgG2a titers increased significantly after vaccinations (*p* < .05). However, the increase in serum IgG2a titers was considerably higher in mice vaccinated with Alhydro gel plus MPLA as compared to those immunized with Alhydrogel only (*p* < .05) (Figure 4B).

**Figure 5:**
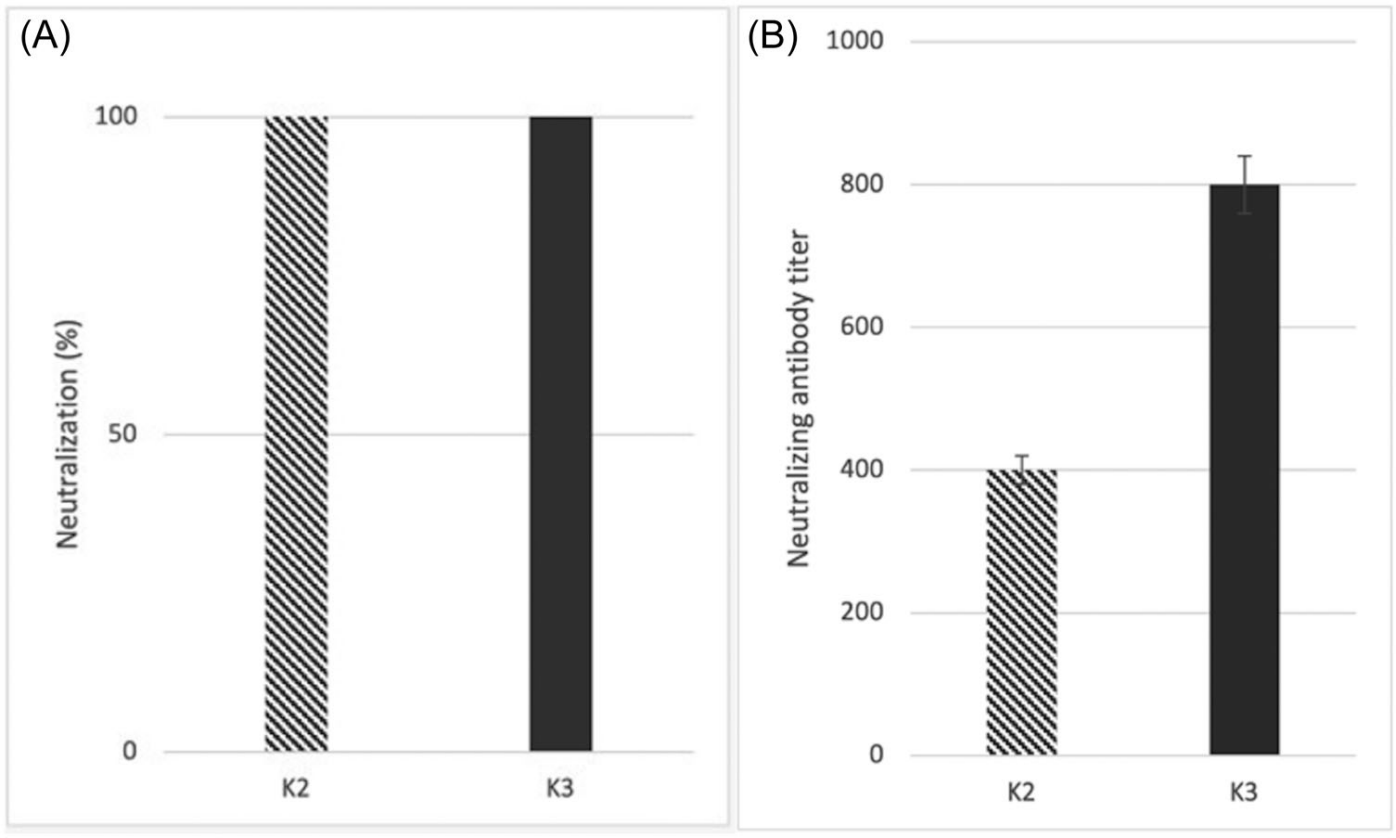
(A) SARS‐CoV‐2 wild virus neutralization test of mouse sera immunized with recombinant P1 + P2 + N vaccine formulations with either Alhydrogel (K2) or Alhydrogel + MPLA (K3) as an adjuvant. Neutralization capacity (NT100) was expressed as being able to completely inhibit virus‐induced CPEs in 100% of the wells. (B) Neutralization of P1 (containing RBD) protein–ACE2 interaction. ACE‐2, angiotensin‐converting enzyme 2; CPE, cytopathic effect; MPLA, monophosphoryl lipid A; RBD, receptor‐binding domain; SARS‐CoV‐2, severe acute respiratory syndrome coronavirus 2.

These formulations also induced high virus neutralization (Figure 5A,B) and elicited cell‐mediated immunity, as demonstrated by enhanced INF‐γ production by immunized Mouse splenocytes (Figure 4C).

## 4 DISCUSSION

Pollet et al. discussed that the first‐generation SARS‐CoV‐2 vaccines based on DNA, RNA, and viral vector‐based strategies attracted more interest and funding at the beginning of the pandemic. They are brought to clinical use much faster than recombinant protein vaccines.^12^ Except for the inactivated whole‐virion SARS‐CoV‐2 vaccines like CoronaVac® and the recombinant subunit vaccines, including full‐length S‐protein trimer Novavax® and RBD dimer ZF2001® produced by insect and mammalian cell lines, respectively, all of the vaccines approved for COVID‐19 emergency use today are either mRNA or viral vector vaccines. For combating COVID‐19 worldwide, the efforts for the development of much safer, more effective, and inexpensive vaccines are needed not only for adults but for children, pregnant women, and the elderly as well, and also to achieve global vaccine equity considering low‐income countries. The success in the fight against the ongoing pandemic is also linked to other factors, including pathogen adaptation and waning immunity which carry reservations in terms of both antigenic diversity and reactogenicity. There remains much to be learned about the long‐term performance of the first‐generation vaccines in use, the exact duration of protection provided by them, their side effects, and their suitability for quick modification and optimization against new variants of SARS‐CoV‐2.

Full spike or RBD protein has been the most critical focus for the development of SARS‐CoV‐2 vaccines and diagnostics and therapeutic antibodies. However, being very large, metastable, and heavily glycosylated, it is challenging to produce a whole prefusion‐stabilized spike by recombinant technology.^13^, ^14^ There have been structural studies, adjuvant approaches, and protein engineering strategies to increase the stability of prefusion S‐protein antigen.^13^, ^15^, ^16^, ^17^

Several expression systems such as mammalian, plant, and insect cells, yeasts and bacteria have been employed and reported for recombinant expression of the S1 region or solely the RBD domain.^18^, ^19^, ^20^, ^21^, ^22^ Commercialized recombinant protein vaccines with their safety and more controllable risk factors are thought to offer advantages over those with a limited history of clinical use. Among the alternative expression hosts, prokaryotic expression systems, *E. coli* in particular, have been preferred for the availability of several molecular manipulation tools, time‐cost saving, and ease in scaling‐up for manufacture, hence suitability for mass production.^23^, ^24^ *E. coli* expression systems have long been proved their potential, recalling the efficacy of the former licensed subunit vaccines, including those against hepatitis E virus, human papillomavirus, and meningococcal B infections.^12^, ^24^, ^25^

The use of the *E. coli* expression system in this study was also very successful in protein yield, antigenic quality, and stability (data not shown).

While RBD in S1 subunit is the main target of most nAbs against SARS‐CoV‐2, it has been reported that T cell responses to S and N proteins are the most dominant and prolonged, especially among structural proteins. Particularly cytotoxic T cells (CTL) play a crucial role in clearing respiratory viruses and can provide long‐term protective cellular immunity.^26^, ^27^, ^28^ Studies with acute and convalescent COVID‐19 patients have demonstrated that T cell responses are associated with reduced disease, suggesting that SARS‐CoV‐2‐specific CD4+ T cell and CD8+ T cell responses are essential for the control and resolution of primary SARS‐CoV‐2 infection.^7^ At the same time, it is known that the presence of N protein in SARS‐CoV‐2 vaccines is of great importance for the T cell response.^29^, ^30^

We developed two antigenic critical fragment proteins of the S1 and S2 regions in the vaccine formulations. The whole N protein was recombinantly produced and formulated with alum or alum + MPLA adjuvant. In this study, the P1 protein containing the RBD region, the P2 protein including the critical T cell epitopes of the S2 region, and the whole N protein showed strong antigenic reactions with the recovered patient sera. At the same time, we observed that our P2 fragment also included T cell epitopes predicted by Grifoni et al.^31^ via sequence homology and bioinformatics approaches induced specific high IgG2a and INF‐γ levels.

Our findings also showed that both adjuvant formulations of our P1 + P2 + N subunit vaccine are pretty successful in terms of the humoral and cellular responses they induced. In our formulation adsorbed onto alum adjuvant, the IgG2a level was even higher for P2 than for the P1 component of the vaccine. While high IgG and IgG2a titers were observed against antigens in mice immunized with both formulations, IgG2a and specific IFN‐γ responses were higher in mice immunized with the Alum + MPLA formulation. In addition, the formulation containing Alum + MPLA induced a much better Th1 response, especially when P2 and N antigens were considered.

MPLA, as a TLR4 agonist molecule, has been included in approved human vaccines after formulation with liposomes, oil emulsions, or aluminum salts. TLR4, which is expressed on the plasma membrane of human macrophages and dendritic cells, is engaged by the bacterial lipopolysaccharide, MPLA, and synthetic derivative. MPLA induces the production of IgG2a antibodies and supports the generation of Th1 immunity.^32^, ^33^ Human vaccine trials indicate that MPLA has a safety profile similar to alum. Extensive clinical studies were conducted using MPL® (also known as AS04), consisting of MPLA absorbed onto aluminum hydroxide or aluminum phosphate. The combination of AS04 + hepatitis B vaccine (FENDrix®) was studied in immune‐compromised individuals (including the elderly with immune deficiency diseases and patients on hemodialysis). FENDrix® was well tolerated and induced higher seroprotection rates and Ab titers than Engerix‐B® (the licensed HBV vaccine) in multiple clinical studies.^34^, ^35^

Knowing the innate and adaptive immunity level against SARS‐COV‐2 infection is crucial. Understanding the short and long‐term immune mechanisms after viral infection is significant because this will create implications that will ensure the protection and continuity of the vaccines to be developed. However, it has been noted that an effective CD4+ and CD8+ T cell response is protective against SARS‐CoV‐2. Still, it is challenging to generate an early response in humans due to the effective innate immune‐avoidance mechanisms of SARS‐CoV‐2. Immunity avoidance caused by SARS‐CoV‐2 is exacerbated by decreased antigen‐presenting cell (APC) function in the elderly.^36^

In conclusion, the findings obtained in this study show that both alum and Alum + MPLA formulations are incredibly successful in terms of humoral and cellular responses. However, since the Alum + MPLA formulation appears superior in terms of Th1 response, we think this vaccine candidate can be recommended mainly for the elderly and immune‐compromised individuals and patients with immunodeficiency diseases on hemodialysis. We also believe that the alum‐only formulation will provide great benefits for adults, young adolescents, and children. These issues will be evaluated in detail in the phase studies implemented.

In addition, this formulation model can be quickly adapted to include important variant proteins. In our ongoing work in this direction, we aimed at adding the Omicron RBD fragment (POm; 33 kDa) to our vaccine candidate. Currently, Omicron RBD has been obtained by the same method and its preclinical study is underway.

## AUTHOR CONTRIBUTIONS

Erkan Özcengiz designed the research study. Erkan Özcengiz and Gülay Özcengiz. planned the experiments. Erkan Özcengiz, Duygu Keser, Gözde Çelik, and Fatma Nur İnçeh performed the experiments. Aykut Özkul carried out VNT assays. Erkan Özcengiz and Gülay Özcengiz wrote the manuscript. All authors have read and agreed to the manuscript.

## CONFLICT OF INTEREST

The authors declare no conflict of interest.

## ETHICS STATEMENT

All animal experiments were performed with the approval of the Ethics Committee on Animal Experimentation, Kobay A.Ş., Ankara, Turkey.

## Data Availability

Data sharing not applicable to this article as no datasets were generated or analyzed during the current study.
